# Dark-Induced Hormonal Regulation of Plant Growth and Development

**DOI:** 10.3389/fpls.2020.581666

**Published:** 2020-10-07

**Authors:** Deepika, Ankit, Sushma Sagar, Amarjeet Singh

**Affiliations:** National Institute of Plant Genome Research, New Delhi, India

**Keywords:** dark, signaling, hormone, growth, development

## Abstract

The sessile nature of plants has made them extremely sensitive and flexible toward the constant flux of the surrounding environment, particularly light and dark. The light is perceived as a signal by specific receptors which further transduce the information through the signaling intermediates and effector proteins to modulate gene expression. Signal transduction induces changes in hormone levels that alters developmental, physiological and morphological processes. Importance of light for plants growth is well recognized, but a holistic understanding of key molecular and physiological changes governing plants development under dark is awaited. Here, we describe how darkness acts as a signal causing alteration in hormone levels and subsequent modulation of the gene regulatory network throughout plant life. The emphasis of this review is on dark mediated changes in plant hormones, regulation of signaling complex COP/DET/FUS and the transcription factors PIFs which affects developmental events such as apical hook development, elongated hypocotyls, photoperiodic flowering, shortened roots, and plastid development. Furthermore, the role of darkness in shade avoidance and senescence is discussed.

## Introduction

Light and dark, both act as environmental cues that regulate plant growth and development from seedling emergence till senescence. Plant development begins in the soil where darkness acts as a signal for etiolation which is characterized by elongated hypocotyl and shortened roots, apical hook of closed cotyledons covering shoot apical meristem and impaired chloroplast development (Arsovski et al., 2012; Mazzella et al., 2014; Gommers and Monte, 2018). This mode of plant growth is known as skotomorphogenesis. It is an evolutionary advanced program in angiosperms to safely get seedling through the soil to light (Seluzicki et al., 2017). Nonetheless, exposure of seeds to continuous light during early development causes seedling de-etiolation (photomorphogenesis) characterized by attenuated hypocotyl growth, root growth acceleration, apical hook straightening and chloroplast maturation.

Being photoautotrophs, plants have evolved with the diverse set of photoreceptors. *Arabidopsis* photoreceptors have been classified as phytochromes (phyA-E, red/far-red light), cryptochromes (cry1-2), phototropins (phot1-2) and zeitlupe family members (*ZTL*, blue/UV-A light and *UVR8*; UV-B light) (Galvao and Fankhauser, 2015; Mishra and Khurana, 2017; Podolec and Ulm, 2018). Light-mediated activation of different photoreceptors and subsequent release of the dark-mediated photomorphogenic repression leads to light-dependent plant development. Some of the major mechanisms that play important role in photomorphogenic development are phosphorylation of phytochrome interacting factors (PIFs), ubiquitin-mediated proteolysis (UMP), and modulation of CONSTITUTIVE PHOTOMORPHOGENIC/DE-ETIOLATED/FUSCA (COP/DET/FUS) complex activity, organization and subcellular localization of positive regulators of light signaling like ELONGATED HYPOCTYL 5 (HY5), LONG AFTER FAR-RED LIGHT1 (LAF) and LONG HYPOCOTYL AFTER FAR-RED LIGHT1 (HFR1) (Huang et al., 2014; Pham et al., 2018). Dark germinated seedlings become sensitive to fluctuations of the day/night cycles when they are first exposed to light. Therefore, growth and development of seedling comes under the control of circadian clock components. Ample amount of information is available about how plants sense and respond to the complex light spectra, but the information is missing about their behavior under darkness, and light and dark signal interaction. Since, light simply reverses the dark-mediated development by activation of the photoreceptors, it has been speculated that the inactive photoreceptors might act as dark receptors and mediate the dark-triggered signal transduction (Seluzicki et al., 2017; Gommers and Monte, 2018; Armarego-marriott and Sandoval-ibañez, 2020). Nonetheless, exact mechanism of dark sensing and perception is still unknown, and the idea of darkness perception by the inactive light receptors is a matter of debate. Though, regulation of seedling establishment by the light and dark signaling in *Arabidopsis* was recently reviewed (Gommers and Monte, 2018), enough literature is not available for light and dark signal integration and consequent phenotypic alterations.

In this review, an update about how plants make sense of darkness and use it as a signal during different phases of skotomorphogenic development e.g., apical hook formation, hypocotyl elongation, shortened roots, photoperiodic flowering, and plastid development is provided. Moreover, how endogenous clock integrates plant growth and development with photoperiods, and the effect of darkness on the plant responses such as, shade avoidance and dark-mediated senescence is discussed.

## Darkness as a Signal

**FIGURE 1:**
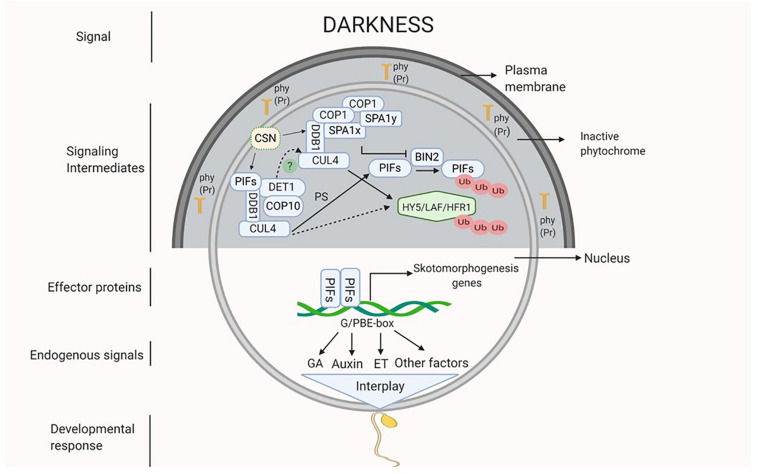
Darkness acts as a signal which might be perceived by inactive phytochromes (Pr form of phy) that activates the signaling intermediates COP1-SPA complex (CUL4-DDB1-COP1-SPA) and CDD complex (CUL4-DDB1-COP10-DET1) which interact with each other (dotted line). Active COP1-SPA complex lead to degradation of HY5 and inactivates BIN2 which causes degradation of PIFs by UMP and active CDD stabilizes PIFs. Active PIFs induces the expression of phytohormones (ET, GA and auxin) biosynthesis and signaling genes and of other skotomorphogenesis signals. Hormonal interplay along with many other factors integrate to generate skotomorphogenic developmental response.

As mentioned earlier, it is presumed that darkness might be perceived by the inactive light receptors that could activate the COP/DET/FUS complex proteins and the PIFs. The proteins of COP/DET/FUS complex are encoded by a group of pleiotropic genes. These proteins are assembled in three different functional complexes, i.e., SUPPRESSOR OF PHYA105 (COPI-SPA), COP10-DET1-DDB1 (CDD), and COP9 signalosome complex (CSN), all of them are connected by a scaffold protein CULLIN4 (CUL4) (Chen et al., 2010; Pokhilko et al., 2011; Dong et al., 2014; Huang et al., 2016). In darkness, COPI-SPA and CDD complex act together to degrade positive regulators of photomorphogenesis like HY5, LAF and HFR1 via UMP. However, these complexes also prevent degradation of PIFs (PIF3 and 4) by inhibiting a brassinosteroid (BR) signaling component BRASSINOSTEROID-INSENSITIVE 2 (BIN2) (Dong et al., 2014; Huang et al., 2016; Wang and Guo, 2018; Figure 1). The function of CSN complex is to derubylate and thus positively regulates the activity of CUL4 which is present on both COP1-SPA and CDD complexes (Chen et al., 2010; Dong et al., 2014). Additionally, DET1 interacts with PIFs under darkness to stabilize them (Dong et al., 2014; Pham et al., 2018). PIFs in turn activate a diverse set of hormone biosynthetic and signaling genes that promote etiolation and repression of photomorphogenic response (Leivar et al., 2008; Paik et al., 2017). In *Arabidopsis*, PIF1, 3, 4, and 5 are involved in skotomorphogenesis, as indicated by normal light-grown seedling like growth of quadruple mutant *pif1pif3pif4pif5* (*pifq)* under complete darkness (Dong et al., 2014; Pfeiffer et al., 2014).

In the forthcoming sections, how darkness regulates development of specific skotomorphogenic structures such as, apical hook, elongated hypocotyls, and shortened roots is discussed.

## Hormonal Regulation of Plant Development in Dark

### Apical Hook Development

Within 24 h of seed germination, after hypocotyl elongation, darkness induces the development of an apical hook (Mazzella et al., 2014) with curvature formation by modulating several hormonal pathways (de Wit et al., 2016). An asymmetrical distribution of auxin (auxin concentration increases at concave side and inhibit cell expansion) causes differential cell expansion and division at both the sides of the hypocotyl. This leads to faster growth at the outer side compared to inner side, which culminates in the hook formation (Béziat and Kleine-Vehn, 2018; Gommers and Monte, 2018; Wang and Guo, 2018). Asymmetric distribution of auxin is facilitated by the influx and efflux carriers on the cell membrane. In *Arabidopsis*, influx carriers AUXIN1 (AUX1) and LIKE AUXIN (LAX3) localize at the epidermal cells and vascular cylinder of the hook. These carriers facilitate the polar and basipetal flow of auxin from shoot apical meristem and cotyledons to hypocotyl. Efflux carrier PIN1 facilitates the outward flow of auxin through vascular cylinder and inner epidermis. PIN3 facilitates the auxin flow from vascular tissues to outer cortex and epidermis, whereas PIN4 and 7 support the auxin flow from vascular tissue to cortex and epidermis. In addition, ABCB transporter 1 and 19 which are localized at inner epidermal cells of the hook, steer the auxin transport to the convex side (Vandenbussche et al., 2010; Wu et al., 2010; Žádníkova et al., 2010, 2016; Farquharson, 2017; Wang and Guo, 2018). The maintenance of differential auxin gradient involves the co-ordination of auxin synthesis, transport and signaling. YUCs and TAR2 are the flavin monooxygenases and tryptophan aminotransferase related enzymes, which, respectively, catalyze two initial steps in the auxin biosynthesis pathway. These genes are differentially expressed in the apical hook region and their mutants (*yuc1/2/4/6, tar2*) display impaired hook phenotype due to developmental defects (Stepanova et al., 2008, 2011; Vandenbussche et al., 2010; Abbas et al., 2013; Cao et al., 2019). The auxin signaling mutants like *iaa3*, *iaa12* and *iaa13* lack apical hook, suggesting the involvement of auxin signaling in apical hook formation. Moreover, AUX/IAAs regulate auxin signaling effector proteins; auxin response factors (ARFs) (Žádníkova et al., 2010, 2016; Abbas et al., 2013).

The apical hook development is tightly regulated by signals from cell wall and root-hypocotyl interaction. The cell wall status affects apical hook bending by transcriptional regulation of *PIN*s and *AUX1* through ARF2 (Aryal et al., 2020; Sampathkumar and Eng, 2020). Recently, it was shown that auxin gradient formed at the root tip by PIN2 is required for root growth in response to gravity (Zhu and Gallem, 2019). The root auxin gradient gradually extends toward hypocotyl and might trigger the hypocotyl bending resulting in hook formation. A balanced concentration of hormones ABA and GA has been shown to be essential for a close interaction between root and hypocotyl (Baral et al., 2020).

PIFs have been involved in different aspects of gradient formation such as, auxin synthesis and polar auxin transport (PAT). In *Arabidopsis*, PIF4 and PIF5 induce the expression of *YUCCA* genes (Pfeiffer et al., 2014) and PIN localization regulatory kinase *WAG2* (Willige et al., 2012; Mazzella et al., 2014). Darkness induced phytohormones such as, GA and ethylene (ET) affect PAT indirectly by modulating the expression of *WAG2* (Willige et al., 2012) and *ETHYLENE INSENSITIVE 3/EIN3-LIKE1* (*EIN3/EIL1*) (Wabnik et al., 2016; Wang and Guo, 2018). PIFs and EIN3/EIL1 co-regulate the curvature formation by directly binding to *HOOKLESS 1* (*HLS1*) promoter. *HLS1*, which codes for a putative N-acetyltransferase and transcriptionally regulated by EIN3/EIL1 is a key regulator of apical hook development (Wang and Guo, 2018). Markedly, PIFs enhance the expression of *HLS1* by binding to its promoter at a site different from that of EIN3/EIL1. In addition, PIFs work in tandem with EIN3/EIL1 to integrate hormonal signals such as, GA, JA, and physical factors including, light and mechanical pressure (Zhang et al., 2018). Besides acting along with PIFs, EIN3 also induces the expression *PIF3* (Zhong et al., 2012). Also, divergent roles of ET in the light- and dark- mediated seedling growth are recently shown (Yu and Huang, 2017; Harkey et al., 2018, 2019; Gu et al., 2019).

In response to darkness, GA accumulates and binds to its receptor gibberellin insensitive dwarf 1 (GID1) and targets DELLAs (GAI, RGA), the negative regulators of GA signaling for UMP degradation (de Lucas et al., 2008). GA is essential for skotomorphogenesis as GA mutant *ga1* seedlings and GA biosynthesis inhibitor Paclobutrazol (PAC) treated seedlings show light-grown phenotype and are unable to form hook even when grown in the complete darkness (Achard et al., 2007; Feng et al., 2008; Arana et al., 2011; Zhang et al., 2018). Antagonistic to auxin, GA promotes cell expansion and division on the convex side of hypocotyl (Alabadí et al., 2008; Arana et al., 2011).

In darkness, ET synthesis is enhanced by the transcriptional activation of *ACS8* by PIF5 in GA dependent manner (Arana et al., 2011). In addition to darkness, physical factors such as, soil depth and compactness, mechanical pressure generated by hypocotyl against soil induce ET production (Zhong et al., 2014; de Wit et al., 2016). ET positively regulates skotomorphogenesis as dark-grown seedlings when treated with exogenous ET, produces exaggerated hooks (Mazzella et al., 2014). Similar to auxin and GA, ET also regulates cell division but at the apical basal parts of the hook. In addition, ET maintains auxin gradient by regulating its synthesis, transport and signaling (An et al., 2012; Chang et al., 2013; Wang et al., 2014; Wabnik et al., 2016; Zhang et al., 2018). JA and SA both are negative regulators of apical hook formation, and both of them act by disrupting ET signaling. JA activated MYC2 promotes the degradation of EIN3/EIL1. Also, MYC2 and NON-EXPRESSOR OF PR GENES1 (NPR1, SA signaling mediator) individually interact with EIN3 and inhibits its binding to the promoter of *HLS1* (Zhang et al., 2018; Bertoni, 2020; Huang et al., 2020).

### Hypocotyl Elongation

Another remarkable phenotype observed under darkness is an elongated hypocotyl. Darkness leads to this phenotype by modulating the levels of phytohormones such as, auxin, BR, ET, and GA (Reed et al., 2018). Irreversible increase in the plant organ size is primarily caused by cell expansion. Expansion of a cell is characterized by the vacuole enlargement and selective cell wall loosening, which releases the wall pressure and allows the water to flow inside (Cosgrove, 2016a,b). Consistently, the up-regulation of several auxin responsive cell wall loosening related genes such as, *EXPANSIN*s (*EXPA4*,*11*), *EXPANSIN*- *LIKE* (*EXLA3*) and *XYLOGLUCAN ENDOTRANSGLUCOSYLASE/HYDROLASE* (*XTH18*,*19*) was found in dark-grown seedlings (Cosgrove, 2005; Orden et al., 2010; Majda, 2018). Auxin acidifies the wall matrix by stimulating the activity of epidermal cell H^+^-ATPases and K^+^ channels, thereby, generating the turgor pressure (Ivakov et al., 2017; Majda, 2018; Duman et al., 2020). Therefore, cell wall remodeling by auxin and cell wall generated signals promotes hypocotyl elongation. PIFs maintain auxin gradient and induce the expression of wall loosening enzymes (Rougemont et al., 2012; Leivar and Monte, 2014). PIF4 remains functional under darkness by binding directly to BR stabilized protein BZR1 (Wang et al., 2012). Active PIF4-BZR1 module positively regulates the synthesis of GA by targeting DELLAs. Thus, darkness acts through the functional PIF4-BZR1 by regulating the level of phytohormones. ABA has been found to be a negative regulator of hypocotyl elongation in the dark-grown seedlings. ABA induces the expression of DELLAs (*GAI* and *RGA*) and inhibits the expression of auxin biosynthetic genes and membrane H^+^-ATPases (Hayashi et al., 2014; Lorrai et al., 2018).

### Shortened Roots

Like shoots, roots also sense light, consequently, root morphology is altered after light perception (Lee et al., 2017). Dark-germinated seedlings have short and thin primary roots with reduced lateral roots, whereas, the phenotype is reversed after light exposure (Dyachok et al., 2011). So, how the dark signaling represses root growth, and the light perceived at axial end of the plant alters root morphology is a curious question?

**FIGURE 2:**
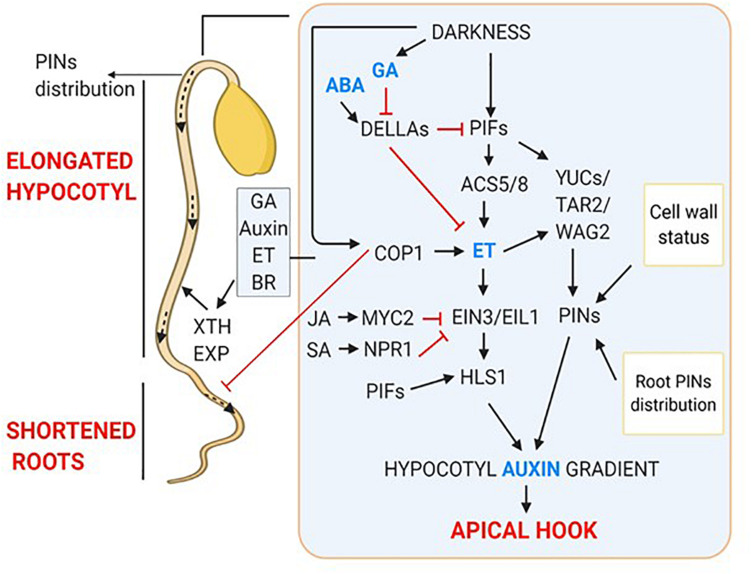
Hormonal regulation of skotomorphogenic organs; apical hook, elongated hypocotyls and shortened roots. Dark-induced signaling results in increased gradient of GA, ET and Auxin which together interplay to form apical hook. Outside inset showing hormonal regulation of hypocotyl elongation and short roots.

When light grown seedlings were decapitated or treated with PAT inhibitors, they showed inhibition of lateral root development (Salisbury et al., 2007), like the etiolated seedlings. This indicated that auxin synthesized in cotyledons in response to light acts as a positive regulator of lateral root emergence. Also, light facilitates auxin transport from apical part of shoot to root (Lee et al., 2016, 2017). In darkness, COP1 inhibits *PIN1* gene expression in shoot. PIN1 is essential for shoot to root PAT therefore, repression of its expression leads to the root growth suppression. But, when shoot is exposed to light, COP1 moves out from the nucleus, relieving the suppression of *PIN1* gene expression (Sassi et al., 2012; Gangappa and Botto, 2016). The HY5 deficient mutant also exhibits defects in lateral root elongation and growth, suggesting the involvement of HY5 in maintaining shoot to root continuum (Sibout et al., 2006). Light activates photoreceptors which interact with COP1, leading to its inactivation. Light-dependent inactivation of COP1 inhibits COP1 mediated degradation of HY5 thereby, promoting HY5 activity. Interestingly, light stabilized HY5 targets COP1 for degradation, thereby, shoot to root PAT is resumed that leads to altered root morphology (Mazzella et al., 2014). Moreover, HY5 translocation from shoot to root promotes root growth (Zhang et al., 2017). The dark-induced hormonal regulation of skotomorphogenic development is summarized in Figure 2.

### Photoperiodic Flowering

Plants sense diurnal variations which affect flowering and accordingly are classified as short- day, long-day and day neutral plants. Importance of darkness in short-day plants is evident from the fact that disruption of dark period with light significantly affects flowering (Andrés and Coupland, 2012; Johansson and Staiger, 2015; Cao et al., 2016). Under long-day condition, light inhibits the expression of flowering genes; *HEADING DATE 3A* (*HD3A*) and *RICE FT-LIKE1* (*RFT1*) in rice by activating an inhibitor HEADING DATE 1 (HD1), whereas, under short-days, HD1 induces the expression of *HD3A* and *RFT1* (Ishikawa et al., 2005). Interruption of long duration of darkness with short exposure of light induces PHOTOPERIOD1 (PPD1) mediated activation of FT1, which after moving from leaves into shoot apical meristem promotes accelerated flowering in wheat (Pearce et al., 2017). Moreover, a night break (NB) causes transcriptional up-regulation of *PPD1* in wheat, levels of which increases with multiple NB and length of darkness. Thus, a period of darkness plays an important role in regulating photoperiodic flowering in plants.

### Light- and Dark-Dependent Plastid Development

Development of plastids could be understood by the studies on the dark-grown seedlings. Plastids could be of various types like, proplastids, eoplasts, etioplasts, chloroplasts and chromoplasts based on their morphological characters, function and tissue location (Liebers et al., 2017). In the cotyledon of the dark-grown seedlings, eoplasts develop into etioplast and at this stage, the development of a prolamellar body (PLB) occurs (Bastien et al., 2016). A PLB is made up of regular arrangements of NADPH, the chlorophyll precursor protochlorophyllide (Pchlide), protochlorophyllide-oxido-reductase (POR), and the thylakoid membrane lipids monogalactosyldiacylglycerol (MGDG) and digalactosyldiacylglycerol (DGDG). In light, various nuclear genes which code for chloroplast biogenesis related proteins are expressed. This results in thylakoid membrane development and POR induced chlorophyll biosynthesis. The proplastids in the shoot apical meristem are directly converted to chloroplast during primary leaf development (Liebers et al., 2017).

### Molecular Aspects of Plastid Development

TFs involved in early developmental process regulate the expression of plastid development related genes. PIF1 and PIF3 accumulate in dark-grown seedlings and repress the chlorophyll biosynthesis genes, whereas, in response to red light PIFs undergo active phy (Pfr) mediated degradation (Gommers and Monte, 2018; Hernández-Verdeja et al., 2020). Similarly, in light, EIN3 is directly targeted and degraded by crys and phys (Gommers and Monte, 2018).

In an early response to light the expression of *HY5*, which regulates the downstream components of chloroplast development, is induced. In dark COP1 mediates the degradation of HY5 and stabilization of PIF and EIN3. Under blue light, cryptochrome represses the expression of *COP1* leading to enhanced HY5 activity, which is required for chloroplast development (Hernández-Verdeja et al., 2020). PIFs and HY5 act as negative and positive regulators, respectively, of chlorophyll and carotenoid synthesis. Therefore, PIFs and HY5 compete for the same binding site (G-box) on the promoter of common target genes, and eventually they regulate chloroplast development antagonistically (Toledo-Ortiz et al., 2014). In addition, HY5-PIF module controls the photosynthetic gene transcription by regulating *PHYTOENE SYNTHASE* (*PSY*) gene, which catalyzes a rate limiting step in carotenoid biosynthesis pathway (Toledo-Ortiz et al., 2014). Thus, the PIF-HY5 regulatory mechanism is crucial for the proplastid development. Golden2-Like (GLK) nuclear TFs which work independently of phytochromes and PIFs are also the key regulators of plastid biogenesis (Liu et al., 2020). GLKs strongly activate the chlorophyll biosynthesis enzymes and light-harvesting chlorophyll binding proteins including, GlutRNA reductase (HEMA1), magnesium chelatase (CHLH), pchlide oxidoreductase (POR-B) and chlidea oxygenase (CAO).

Two RNA polymerases; nuclear encoded RNA polymerase (NEP) and plastid encoded RNA polymerases (PEP) are involved in the proplastid biogenesis (Hernández-Verdeja et al., 2020). In dark, plastid gene transcription is driven by NEP whereas, upon light exposure, transition of NEP to PEP occurs leading to PEP driven transcription. In light, the main transcriptional activity is taken-up by PEP, thus, the transcription of PEP associated genes increases. PRIN2, a plastid localized redox regulated protein is required for PEP activity. In dark, PRIN2 forms a homo-dimer via di-sulfide bonds, however, upon exposure to light it get reduced to PRIN2 monomers, and contributes to PEP activity (Díaz et al., 2018). PEP mediated increase in transcription and light induced communication between developing chloroplast and nucleus through antero-retrograde signaling results in completion of chloroplast biogenesis (Pogson et al., 2015).

### Hormonal Regulation of Plastid Development

Some phytohormones control the chlorophyll biosynthesis, thereby, regulate the chloroplast biogenesis. For example, in the dark-grown *Arabidopsis* seedlings cytokinin treatment induces the formation of prothylakoids whereas, these thylakoid membranes developed after about 6 h of light treatment in cytokinin untreated seedlings (Cortleven and Schmülling, 2015). The chlorophyll biosynthesis begins with the conversion of glutamate to 5-aminolevulinic acid (ALA), followed by the production of chlorophyll precursor, Pchlide. POR catalyzes the conversion of Pchlide into chlorophyllide which after esterification forms chlorophyll. Cytokinin promotes ALA synthesis and enhances POR activity to support the chloroplast biogenesis (Liu et al., 2017). GA regulated DELLA proteins are involved in the induction of *PORA* and *PORB* genes (Cheminant et al., 2011). Involvement of ET signaling has also been found in plastid development. In the double mutant of ET signaling; *ein3eil1* expression of Pchlide/chlorophyll synthesis genes *HEMA1*, *GUN4*, and *GUN5* was increased under darkness. PIF3 has been known to inhibit the expression of these genes (Stephenson et al., 2009; Shin et al., 2009), consistently, their expression was higher in *pif3* mutant. Moreover, *ein3eil1mutant* but not *pif3* had reduced expression of *PORA* and *PORB* genes. Thus, EIN3/EIL1 negatively regulate Pchlide/chlorophll biosynthesis through PIF3, but stimulate *PORA* and *PORB* independently of PIF3 (Zhong et al., 2014). These observations clearly suggest the crucial role of phytohormones in plastid development.

### Shade Avoidance: An Intermediate Response Between Light and Dark

Due to their sessile nature, plants face shade arising from the neighboring vegetation canopies. In the shade, plants compete for their resources particularly red/far-red (R:FR) photon flux ratio. In response to shade, plants channelize their energy toward hypocotyl and stem elongation, enhanced apical dominance and early flowering, all of them collectively termed as shade avoidance syndrome (SAS) (de Wit et al., 2016). Along with photoreceptors, phytohormones like ET, GA, Auxin, and BR are implicated in shade-induced plant responses (Das et al., 2016; Yang and Li, 2017).

In the past decade, shade related research was carried out mainly in *Arabidopsis*, and indicated a crucial role of auxin in the shade avoidance response (Sessa et al., 2018). In the *Arabidopsis* seedlings, auxin accumulates in response to shade resulting in hypocotyl cells elongation (Ma and Li, 2019). A low R:FR ratio (as in case of shade) has been found to promote petiole elongation in *Arabidopsis* (Roig-Villanova and Martínez-García, 2016). Phytochrome B (phyB) is the key shade avoidance response regulating photoreceptor, whereas, phyD and phyE function redundantly in promoting shade-induced elongation (Franklin et al., 2003). In contrast, phyA represses the elongation response induced by low R/FR light (Martínez-García et al., 2014). phyB becomes active in the presence of light having high R:FR ratio. Upon activation, it translocates into the nucleus and interacts with PIFs. This interaction causes phosphorylation and subsequent inactivation of PIFs leading to their degradation via UMP (de Wit et al., 2016). Hence, PIF-dependent transcriptional activation of auxin homeostasis and cell wall remodeling related genes is inhibited (Courbier and Pierik, 2019). While, under light of low R:FR, phyB remains inactive, thus allowing PIFs to accumulate and induce the transcription of *YUCCA* genes (Müller-Moulé et al., 2016), resulting in enhanced auxin concentration in the cell. PIF4, 5, and 7 directly regulate several auxin biosynthesis and signaling genes in response to shade (Hornitschek et al., 2012; Li et al., 2012). A HomeoBox2 (ATHB2) TF acts as a positive regulator and Long Hypocotyl in Far-red 1/Slender In Canopy Shade 1 (HFR1/SICS1) (an atypical bHLH protein), acts as a negative regulator of PIFs controlled shade avoidance response (Sessa et al., 2018). Cryptochromes (cry1 and cry2) are involved in low blue light (LBL)-induced shade avoidance response. CRY1 and CRY2 physically interact with PIFs and regulates their activity for LBL induced hypocotyl growth (Ma et al., 2016; Sessa et al., 2018).

### Phytohormones in Shade Avoidance Response

Upon low R: FR exposure, bioactive GA levels increases and the accumulated GA inhibits the DELLAs. The DELLA proteins directly interact with PIFs and this interaction prevents PIFs binding to DNA, thereby, negatively regulating the expression of cell elongation related genes (Li et al., 2016). Moreover, GA is implicated in shade-induced flowering, as silencing *GA20ox2* expression results in delayed flowering in *Arabidopsis* under far-red light conditions (Yang and Li, 2017). In addition, ET is proposed to be a positive regulator of shade-induced petiole elongation, and is involved in organ specific shade avoidance response (Yang and Li, 2017). ABA biosynthesis mutants, *nced3-2* and *aba2-1* show increased branching under low R:FR suggesting that ABA suppresses branching under shade (Yang and Li, 2017). NPR1 also plays a crucial role in petiole elongation in shade (Nozue et al., 2018). Interestingly, elevated auxin and BR production in response to PIFs activation under low R:FR light costs both SA and JA based defenses (Martínez et al., 2018).

### Day-Night Transitions

The rhythmic behavior of the biological processes is maintained by an endogenous oscillator/pacemaker called the circadian clock. This clock, under natural conditions maintains a period of 24 h, regulated by transitions from day to night (light/dark) and vice versa. As the clock is tightly coupled with diurnal cycles, it modulates many gene regulatory networks (GRNs) based on time of the day. When an organism is subjected to constant light or dark for a longer time, the rhythms dampen out and require transition from the existing environment. Since, plant life begins under complete darkness, the seedling growth during this period is clock independent, till it experiences light/dark (photoperiod) cycles (Salome et al., 2008). The fact that *Arabidopsis* seedling growth becomes photoperiod sensitive after de-etiolation was established by growing plants in continuous light and dark, separately. Until 12 h, similar hypocotyl length was observed in both conditions, and only prolonged exposure of darkness (> 12 h) resulted in elongated hypocotyls, suggesting the process to be short-day specific (Niwa et al., 2009; Seluzicki et al., 2017). Then, how clock is integrated with photoperiod after de-etiolation, was disclosed by the growth pattern of WT and clock mutants (*CCA1*-ox and *elf3*) seedlings in short-day conditions. WT seedlings elongated normally after prolonged darkness, but in both the clock mutants seedling elongation started at the beginning of the dark period and continued till it prolonged, indicating an inhibitory effect of clock genes during initial hours of darkness (Fankhauser et al., 2007). Interestingly, temporal transcriptional induction of *PIF1, 3, 4*, and *5* was also observed during late-night hours coinciding with the seedling etiolation (Leivar et al., 2012). PIFs are degraded during the day through PHY mediated photobodies, and are kept in check during early-night by clock evening complex (EC) genes (Huang et al., 2016). Another clock complex gene, *TIMING OF CAB EXPRESSER 1* (*TOC1*) represses PIFs level during early- and mid-night (Soy et al., 2016; Paik et al., 2017). Therefore, clock exerts its effect by controlling the temporal expression of *PIF* genes under darkness.

### Dark-Induced Senescence

Senescence is an age triggered developmental process characterized by an ordered and programmable degradation. The degradation involves the mobilization of building blocks at various levels of organization e.g., cell, tissue, organ and the organism, culminating in plant death (Lim et al., 2007). Its initiation, progression and completion are tightly linked to various external and internal cues (Kim et al., 2016; Law et al., 2018). Darkness is one of the external cues that positively regulate leaf senescence, and the process is called dark-induced senescence (DIS). DIS is physiologically quite different from an age-triggered leaf senescence (Kiddle et al., 2011). Interestingly, darkness only promotes senescence of individual plant organs, and inhibits senescence at the whole plant level (Keech et al., 2010; Sakuraba et al., 2014; Law et al., 2018), suggesting the involvement of other factors for death of the whole plant.

PIFs involvement in DIS became evident when PIF single and quadruple (*pifq*) mutants exhibited delayed leaf senescence, and their overexpressing plants showed the opposite phenotype (Song et al., 2014). Moreover, *PIF3, 4* and *5* were significantly up-regulated in both age triggered senescence and DIS. Evidences indicate that PIFs mediate transcriptional activation of many SENESCENCE ASSOCIATED GENES (SAGs), i.e., *STAY GREEN 1* (*SGR1*) and *NON-YELLOW COLORING1* (*NYC1*) and other senescence promoting TFs such as *WRKY22* and *NAC* (Zhang et al., 2015). Additionally, PIF4/5 have been shown to transcriptionally enhance the expression of *ABSCISIC ACID 5* (*ABI5*) and *ENHANCED EM LEVELS* (*EEL*) (Qi et al., 2020). *ABI5, EEL*, and PIFs act together in a coherent feed forward loop to increase the expression of *ORESARA1* (*ORE1*), a master regulator of senescence. Subsequently, PIFs, ORE1, ABI5, and EIN3 interplay to activate SAGs which finally lead to breakdown of chlorophyll, degradation of photosynthetic machinery culminating in leaf senescence (Sakuraba et al., 2014; Qiu et al., 2015; Liebsch and Keech, 2016).

## Conclusion and Future Directions

Dark and light independently activate diverse signaling pathways which alter the levels of plant growth regulators consequently leading to a specific response. Efforts made in the past two decades in the area of skotobiology have advanced our understanding of how plant behaves and make sense of the dark period. The role of multifunctional dark signaling intermediates COP/DET/FUS and transcription factor PIF has been explored in diverse areas. We have provided the latest information about darkness acting as a signal during various plant growth processes such as, skotomorphogenesis, day-night transitions, shade avoidance, and senescence. Differential accumulation of several phytohormones, their regulatory effects on diverse molecular components and, in turn, the interplay of molecular players that determines the pattern of growth and development in dark has been elaborated. However, a complete understanding of the dark and light signaling integration needs exploration of the inter-organ communication mechanisms, necessary for establishment/transfer of hormonal gradients. Also, the hormonal interplay and regulatory mechanism underlying the integration of other subterranean environmental cues such as, soil compactness, temperature, biotic and abiotic factors with dark signaling, is still enigmatic and requires in-depth exploration in future.

## Author Contributions

AS conceptualized and designed the study. Deepika, Ankit, and SS compiled the data. Deepika, Ankit, and AS wrote the manuscript. All authors read and approved the final version of the manuscript.

## Conflict of Interest

The authors declare that the research was conducted in the absence of any commercial or financial relationships that could be construed as a potential conflict of interest.

## References

[B1] Abbas M. Alabadí D. Blázquez M. A. (2013). Differential growth at the apical hook: all roads lead to auxin. *Front. Plant Sci.* 4:441. 10.3389/fpls.2013.00441 24204373 PMC3817370

[B2] Achard P. Liao L. Jiang C. Desnos T. Bartlett J. Fu X. (2007). DELLAs contribute to plant photomorphogenesis. *Plant Physiol.* 143 1163–1172. 10.1104/pp.106.092254 17220364 PMC1820925

[B3] Alabadí D. Gallego-Bartolomé J. Orlando L. García-Cárcel L. Rubio V. Martínez C. (2008). Gibberellins modulate light signaling pathways to prevent Arabidopsis seedling de-etiolation in darkness. *Plant J.* 53 324–335. 10.1111/j.1365-313X.2007.03346.x 18053005

[B4] An F. Zhang X. Zhu Z. Ji Y. He W. Jiang Z. (2012). Coordinated regulation of apical hook development by gibberellins and ethylene in etiolated Arabidopsis seedlings. *Cell Res.* 22 915–927. 10.1038/cr.2012.29 22349459 PMC3343656

[B5] Andrés F. Coupland G. (2012). The genetic basis of flowering responses to seasonalcues. *Nat. Publ. Gr.* 13 627–639. 10.1038/nrg3291 22898651

[B6] Arana V. Vandenbussche F. Petra Z. Gallego-bartolome J. Guardiola V. Van Der Straeten D. (2011). Hierarchy of hormone action controlling apical hook development in Arabidopsis. *Plant J.* 67 622–634. 10.1111/j.1365-313X.2011.04621.x 21535259

[B7] Armarego-marriott T. Sandoval-ibañez O. (2020). Beyond the darkness: recent lessons from etiolation and de-etiolation studies. *JXB* 71 1215–1225. 10.1093/jxb/erz496 31854450 PMC7031072

[B8] Arsovski A. A. Galstyan A. Guseman J. M. Nemhauser J. L. (2012). Photomorphogenesis. *Arabidopsis Book* 10:e01475. 10.1199/tab.0147 22582028 PMC3350170

[B9] Aryal B. Jonsson K. Baral A. Sancho-andres G. Kierzkowska A. R. Kierzkowski D. (2020). Interplay between cell Wall and Auxin mediates the control of differential cell elongation during apical hook development. *Curr. Biol* 30 1733–1739.e3. 10.1016/j.cub.2020.02.055 32197084

[B10] Baral A. Morris E. Aryal B. Jonsson K. Verger S. Xu T. (2020). External mechanical cues reveal core molecular pathway behind tissue bending in plants. *bioRxiv* [Preprint]. 10.1101/2020.03.05.978296 33434527

[B11] Bastien O. Botella C. Chevalier F. Block M. A. Jouhet J. Breton C. (2016). New insights on thylakoid biogenesis in plant cells. *Int. Rev. Cell Mol. Biol.* 323 1–30. 10.1016/bs.ircmb.2015.12.001 26944617

[B12] Bertoni G. (2020). Ethylene versus salicylic acid in apical hook formation. *Plant Cell* 32:531. 10.1105/tpc.20.00031 31932486 PMC7054040

[B13] Béziat C. Kleine-Vehn J. (2018). The road to auxin-dependent growth repression and promotion in apical hooks. *Curr. Biol.* 28 R519–R525. 10.1016/j.cub.2018.01.069 29689235

[B14] Cao K. Cui L. Ye L. Zhou X. Bao E. Zhao H. (2016). Effects of red light and night break treatments on growth and flowering of tomato plants. *Front. Plant Sci.* 7:527. 10.3389/fpls.2016.00527 27148344 PMC4840390

[B15] Cao M. Chen R. Li P. Yu Y. Zheng R. Ge D. (2019). Differential growth of the apical hook. *Nature* 568 240–243. 10.1038/s41586-019-1069-7 30944466

[B16] Chang K. N. Zhong S. Weirauch M. T. Hon G. Pelizzola M. Li H. (2013). Temporal transcriptional response to ethylene gas drives growth hormone cross-regulation in Arabidopsis. *eLIFE* 2 e00675. 10.7554/eLife.00675 23795294 PMC3679525

[B17] Cheminant S. Wild M. Bouvier F. Pelletier S. Renou J. P. Erhardt M. (2011). DELLAs regulate chlorophyll and carotenoid biosynthesis to prevent photooxidative damage during seedling deetiolation in Arabidopsis. *Plant Cell* 23 1849–1860. 10.1105/tpc.111.085233 21571951 PMC3123943

[B18] Chen H. Huang X. Gusmaroli G. Terzaghi W. Lau O. S. Yanagawa Y. (2010). Arabidopsis CULLIN4-Damaged DNA Binding Protein 1 Interacts with CONSTITUTIVELY PHOTOMORPHOGENIC1-SUPPRESSOR OF PHYA Complexes to Regulate Photomorphogenesis and Flowering Time. *Plant Cell* 22 108–123. 10.1105/tpc.109.065490 20061554 PMC2828697

[B19] Cortleven A. Schmülling T. (2015). Regulation of chloroplast development and function by cytokinin. *J. Exp. Bot.* 66 4999–5013. 10.1093/jxb/erv132 25873684

[B20] Cosgrove D. J. (2005). Growth of the plant cell wall. *Nat. Rev. Mol. Cell Biol.* 6 850–861. 10.1038/nrm1746 16261190

[B21] Cosgrove D. J. (2016a). Catalysts of plant cell wall loosening. *F1000Res*. 5:119. 10.12688/f1000research.7180.1 26918182 PMC4755413

[B22] Cosgrove D. J. (2016b). Plant cell wall extensibility: connecting plant cell growth with cell wall structure, mechanics, and the action of wall modifying enzymes. *JXB* 67 463–476. 10.1093/jxb/erv511 26608646

[B23] Courbier S. Pierik R. (2019). Canopy light quality modulates stress responses in plants. *iScience* 22 441–452. 10.1016/j.isci.2019.11.035 31816531 PMC6909002

[B24] Das D. St. Onge K. R. Voesenek L. A. C. J. Pierik R. Sasidharan R. (2016). Ethylene- and shade-induced hypocotyl elongation share transcriptome patterns and functional regulators. *Plant Physiol.* 172 718–733. 10.1104/pp.16.00725 27329224 PMC5047086

[B25] de Lucas M. Davière J. M. Rodríguez-Falcón M. Pontin M. Iglesias-Pedraz J. M. Lorrain S. (2008). A molecular framework for light and gibberellin control of cell elongation. *Nature* 451 480–484. 10.1038/nature06520 18216857

[B26] de Wit M. Galvão V. C. Fankhauser C. (2016). Light-mediated hormonal regulation of plant growth and development. *Annu. Rev. Plant Biol.* 67 513–537. 10.1146/annurev-arplant-043015-112252 26905653

[B27] Díaz M. G. Hernández-Verdeja T. Kremnev D. Crawford T. Dubreuil C. Strand Å. (2018). Redox regulation of PEP activity during seedling establishment in *Arabidopsis thaliana*. *Nat. Commun.* 9:50. 10.1038/s41467-017-02468-2 29298981 PMC5752674

[B28] Dong J. Tang D. Gao Z. Yu R. Li K. He H. (2014). Arabidopsis DE-ETIOLATED1 represses photomorphogenesis by positively regulating phytochrome-interacting factors in the dark. *Plant Cell* 26 3630–3645. 10.1105/tpc.114.130666 25248553 PMC4213169

[B29] Duman Z. Eliyahu A. Abu-abied M. Sadot E. (2020). The contribution of cell wall remodeling and signaling to lateral organs formation. *Isr. J. Plant Sci.* 67 110–127. 10.1163/22238980-20191115

[B30] Dyachok J. Zhu L. Liao F. He J. Huq E. Blancaflor E. B. (2011). SCAR mediates light-induced root elongation in arabidopsis through photoreceptors and proteasomes. *Plant Cell* 23 3610–3626. 10.1105/tpc.111.088823 21972261 PMC3229138

[B31] Fankhauser C. Nozue K. Covington M. F. Duek P. D. Harmer S. L. Maloof J. N. (2007). Rhythmic growth explained by coincidence between internal and external cues. *Nature* 448 358–361. 10.1038/nature05946 17589502

[B32] Farquharson K. (2017). Division of labor during apical hook formation. *Plant Cell* 29 917–918. 10.1105/tpc.17.00357 28487410 PMC5466039

[B33] Feng S. Martinez C. Gusmaroli G. Wang Y. Zhou J. Wang F. (2008). Coordinated regulation of *Arabidopsis thaliana* development by light and gibberellins. *Nature* 451 475–479. 10.1038/nature06448 18216856 PMC2562044

[B34] Franklin K. A. Praekelt U. Stoddart W. M. Billingham O. E. Halliday K. J. Whitelam G. C. (2003). Phytochromes B, D, and E act redundantly to control multiple physiological responses in arabidopsis. *Plant Physiol.* 131 1340–1346. 10.1104/pp.102.015487 12644683 PMC166893

[B35] Galvao V. C. Fankhauser C. (2015). Sensing the light environment in plants: photoreceptors and early signaling steps. *Curr. Opin. Neurobiol.* 34 46–53. 10.1016/j.conb.2015.01.013 25638281

[B36] Gangappa S. N. Botto J. F. (2016). The multifaceted roles of HY5 in plant growth and development. *Mol. Plant* 9 1353–1365. 10.1016/j.molp.2016.07.002 27435853

[B37] Gommers C. M. M. Monte E. (2018). Seedling establishment: a dimmer switch-regulated process between dark and light signaling. *Plant Physiol.* 176 1061–1074. 10.1104/pp.17.01460 29217596 PMC5813566

[B38] Gu S. Y. Wang L. C. Cheuh C. M. Lo W. S. (2019). CHITINASE like1 regulates root development of dark-grown seedlings by modulating ethylene biosynthesis in *Arabidopsis thaliana*. *Front. Plant Sci.* 10:600. 10.3389/fpls.2019.00600 31156671 PMC6530356

[B39] Harkey A. F. Watkins J. M. Olex A. L. DiNapoli K. T. Lewis D. R. Fetrow J. S. (2018). Identification of transcriptional and receptor networks that control root responses to ethylene. *Plant Physiol.* 176 2095–2118. 10.1104/pp.17.00907 29259106 PMC5841720

[B40] Harkey A. F. Yoon G. M. Seo D. H. DeLong A. Muday G. K. (2019). Light modulates ethylene synthesis, signaling, and downstream transcriptional networks to control plant development. *Front. Plant Sci.* 10:1094. 10.3389/fpls.2019.01094 31572414 PMC6751313

[B41] Hayashi Y. Takahashi K. Inoue S. I. Kinoshita T. (2014). Abscisic acid suppresses hypocotyl elongation by dephosphorylating plasma membrane H+-ATPase in *Arabidopsis thaliana*. *Plant Cell Physiol.* 55 845–853. 10.1093/pcp/pcu028 24492258

[B42] Hernández-Verdeja T. Vuorijoki L. Strand Å. (2020). Emerging from the darkness: interplay between light and plastid signaling during chloroplast biogenesis. *Physiol. Plant.* 169 397–406. 10.1111/ppl.13100 32222991

[B43] Hornitschek P. Kohnen M. V. Lorrain S. Rougemont J. Ljung K. López-Vidriero I. (2012). Phytochrome interacting factors 4 and 5 control seedling growth in changing light conditions by directly controlling auxin signaling. *Plant J.* 71 699–711. 10.1111/j.1365-313X.2012.05033.x 22536829

[B44] Huang H. Alvarez S. Bindbeutel R. Shen Z. Naldrett M. J. Evans B. S. (2016). Identification of evening complex associated proteins in arabidopsis by affinity purification and mass spectrometry. *Mol. Cell. Proteomics* 15 201–217. 10.6019/PXD002606 26545401 PMC4762519

[B45] Huang P. Dong Z. Guo P. Zhang X. Qiu Y. Li B. (2020). Salicylic acid suppresses apical hook formation via NPR1-mediated repression of EIN3 and EIL1 in Arabidopsis. *Plant Cell* 32 612–629. 10.1105/tpc.19.00658 31888966 PMC7054027

[B46] Huang X. Ouyang X. Deng X. W. (2014). Beyond repression of photomorphogenesis: role switching of COP/DET/FUS in light signaling. *Curr. Biol.* 21 96–103. 10.1016/j.pbi.2014.07.003 25061897

[B47] Ishikawa R. Tamaki S. Yokoi S. Inagaki N. Shinomura T. Takano M. (2005). Suppression of the floral activator Hd3a is the principal cause of the night break effect in rice. *Plant Cell* 17 3326–3336. 10.1105/tpc.105.037028.1 16272430 PMC1315372

[B48] Ivakov A. Flis A. Apelt F. Scherer U. Stitt M. Kragler F. (2017). Cellulose synthesis and cell expansion are regulated by different mechanisms in growing Arabidopsis hypocotyls. *Plant Cell* 29 1305–1315. 10.1105/tpc.16.00782 28550150 PMC5502445

[B49] Johansson M. Staiger D. (2015). Time to flower: interplay between photoperiod and the circadian clock. *JXB* 66 719–730. 10.1093/jxb/eru441 25371508

[B50] Keech O. Pesquet E. Gutierrez L. Ahad A. Bellini C. Smith S. M. (2010). Leaf senescence is accompanied by an early disruption of the microtubule network in arabidopsis. *Plant Physiol.* 154 1710–1720. 10.1104/pp.110.163402 20966154 PMC2996031

[B51] Kiddle S. Kim Y. Penfold C. A. Jenkins D. Zhang C. Morris K. (2011). High-resolution temporal profiling of transcripts during arabidopsis leaf senescence reveals a distinct chronology of processes and regulation. *Plant Physiol.* 23 873–894. 10.1105/tpc.111.083345 21447789 PMC3082270

[B52] Kim J. Woo H. R. Nam H. G. (2016). Toward systems understanding of leaf senescence: an integrated multi-omics perspective on leaf senescence research. *Mol. Plant* 9 813–825. 10.1016/j.molp.2016.04.017 27174403

[B53] Law S. R. Chrobok D. Juvany M. Delhomme N. Lindén P. Brouwer B. (2018). Darkened leaves use different metabolic strategies for senescence and survival. *Plant Physiol.* 177 132–150. 10.1104/pp.18.00062 29523713 PMC5933110

[B54] Lee H. Park Y. Ha J. Baldwin I. T. Park C. (2017). Multiple Routes of Light Signaling during Root Photomorphogenesis. *Trends Plant Sci.* 22 803–812. 10.1016/j.tplants.2017.06.009 28705537

[B55] Lee H.-J. Ha J.-H. Kim S.-G. Choi H.-K. Kim Z. H. Han Y.-J. (2016). Stem-piped light activates phytochrome B to trigger light responses in *Arabidopsis thaliana* roots. *Sci. Signal.* 9:ra106. 10.1126/scisignal.aaf6530 27803284

[B56] Leivar P. Monte E. (2014). PIFs: systems integrators in plant development. *Plant Cell* 26 56–78. 10.1105/tpc.113.120857 24481072 PMC3963594

[B57] Leivar P. Monte E. Oka Y. Liu T. Carle C. Castillon A. (2008). Article multiple phytochrome-interacting bHLH transcription factors repress premature seedling photomorphogenesis in darkness. *Curr. Biol.* 18 1815–1823. 10.1016/j.cub.2008.10.058 19062289 PMC2651225

[B58] Leivar P. National S. Sentandreu M. (2012). Phytochrome-imposed oscillations in PIF3 protein abundance regulate hypocotyl growth under diurnal light/dark conditions in Arabidopsis. *Plant J.* 71 390–401. 10.1111/j.1365-313X.2012.04992.x 22409654 PMC3465574

[B59] Li K. Yu R. Fan L. M. Wei N. Chen H. Deng X. W. (2016). DELLA-mediated PIF degradation contributes to coordination of light and gibberellin signalling in Arabidopsis. *Nat. Commun.* 7:11868. 10.1038/ncomms11868 27282989 PMC4906400

[B60] Li L. Ljung K. Breton G. Schmitz R. J. Pruneda-Paz J. Cowing-Zitron C. (2012). Linking photoreceptor excitation to changes in plant architecture. *Genes Dev.* 26 785–790. 10.1101/gad.187849.112 22508725 PMC3337452

[B61] Liebers M. Grübler B. Chevalier F. Lerbs-Mache S. Merendino L. Blanvillain R. (2017). Regulatory shifts in plastid transcription play a key role in morphological conversions of plastids during plant development. *Front. Plant Sci.* 8:23. 10.3389/fpls.2017.00023 28154576 PMC5243808

[B62] Liebsch D. Keech O. (2016). Dark-induced leaf senescence: new insights into a complex light-dependent regulatory pathway. *New Phytol.* 212 563–570. 10.1111/nph.14217 27716940

[B63] Lim P. O. Kim H. J. Nam H. G. (2007). Leaf Senescence. *Annu. Rev. Plant Biol.* 58 115–136. 10.1146/annurev.arplant.57.032905.105316 17177638

[B64] Liu L. Lin N. Liu X. Yang S. Wang W. Wan X. (2020). From chloroplast biogenesis to chlorophyll accumulation: the interplay of light and hormones on gene expression in *Camellia sinensis* cv. Shuchazao Leaves. *Front. Plant Sci.* 11 256. 10.3389/fpls.2020.00256 32218794 PMC7078671

[B65] Liu X. Li Y. Zhong S. (2017). Interplay between light and plant hormones in the control of Arabidopsis seedling chlorophyll biosynthesis. *Front. Plant Sci.* 8:1433. 10.3389/fpls.2017.01433 28861105 PMC5562715

[B66] Lorrai R. Boccaccini A. Ruta V. Possenti M. Costantino P. Vittorioso P. (2018). Abscisic acid inhibits hypocotyl elongation acting on gibberellins. DELLA proteins and auxin. *AoB Plants* 10:ply061. 10.1093/aobpla/ply061 30386544 PMC6204436

[B67] Ma D. Li X. Guo Y. Chu J. Fang S. Yan C. (2016). Cryptochrome 1 interacts with PIF4 to regulate high temperature-mediated hypocotyl elongation in response to blue light. *Proc. Natl. Acad. Sci. U.S.A.* 113 224–229. 10.1073/pnas.1511437113 26699514 PMC4711866

[B68] Ma L. Li G. (2019). Auxin-dependent cell elongation during the shade avoidance response. *Front. Plant Sci.* 10:914. 10.3389/fpls.2019.00914 31354778 PMC6640469

[B69] Majda M. (2018). The role of auxin in cell wall expansion. *Int. J. Mol. Sci.* 19:951. 10.3390/ijms19040951 29565829 PMC5979272

[B70] Martínez C. Espinosa-Ruíz A. Lucas M. Bernardo-García S. Franco-Zorrilla J. M. Prat S. (2018). PIF 4-induced BR synthesis is critical to diurnal and thermomorphogenic growth. *EMBO J.* 37:e99552. 10.15252/embj.201899552 30389669 PMC6276883

[B71] Martínez-García J. F. Gallemí M. Molina-Contreras M. J. Llorente B. Bevilaqua M. R. R. Quail P. H. (2014). The shade avoidance syndrome in Arabidopsis: the antagonistic role of phytochrome A and B differentiates vegetation proximity and canopy shade. *PLoS One* 9:e109275. 10.1371/journal.pone.0109275 25333270 PMC4204825

[B72] Mazzella M. A. Casal J. J. Jorge P. Fox A. R. (2014). Hormonal networks involved in apical hook development in darkness and their response to light. *Front. Plant Sci.* 5:52. 10.3389/fpls.2014.00052 24616725 PMC3935338

[B73] Mishra S. Khurana J. P. (2017). Emerging roles and new paradigms in signaling mechanisms of plant cryptochromes. *CRC Crit. Rev. Plant Sci.* 36 89–115. 10.1080/07352689.2017.1348725

[B74] Müller-Moulé P. Nozue K. Pytlak M. L. Palmer C. M. Covington M. F. Wallace A. D. (2016). YUCCA auxin biosynthetic genes are required for Arabidopsis shade avoidance. *PeerJ* 2016:e2574. 10.7717/peerj.2574 27761349 PMC5068344

[B75] Niwa Y. Yamashino T. Mizuno T. (2009). The circadian clock regulates the photoperiodic response of hypocotyl elongation through a coincidence mechanism in *Arabidopsis thaliana*. *Plant Cell Physiol.* 1 838–854. 10.1093/pcp/pcp028 19233867

[B76] Nozue K. Devisetty U. K. Lekkala S. Mueller-Moulé P. Bak A. Casteel C. L. (2018). Network analysis reveals a role for salicylic acid pathway components in shade avoidance. *Plant Physiol.* 178 1720–1732. 10.1104/pp.18.00920 30348816 PMC6288734

[B77] Orden V. Wolf S. Vissenberg K. Delacourt J. Assoumou Y. Pelletier S. (2010). A role for pectin de-methylesterification in a developmentally regulated growth acceleration in dark- grown Arabidopsis hypocotyls. *New Phytol.* 188 726–739. 10.1111/j.1469-8137.2010.03409.x 20819179

[B78] Paik I. Kathare P. K. Kim J. Huq E. (2017). Expanding roles of PIFs in signal integration from multiple processes. *Mol. Plant* 10 1035–1046. 10.1016/j.molp.2017.07.002 28711729 PMC5551451

[B79] Pearce S. Shaw L. M. Lin H. Cotter J. D. Li C. Dubcovsky J. (2017). Night-break experiments shed light on the photoperiod1-mediated flowering. *Plant Physiol.* 174 1139–1150. 10.1104/pp.17.00361 28408541 PMC5462047

[B80] Pfeiffer A. Shi H. Tepperman J. M. Zhang Y. Quail P. H. (2014). Combinatorial complexity in a transcriptionally centered signaling hub in Arabidopsis. *Mol. Plant* 7 1598–1618. 10.1093/mp/ssu087 25122696 PMC4587546

[B81] Pham V. N. Kathare P. K. Huq E. (2018). Phytochromes and phytochrome interacting factors. *Plant Physiol.* 176 1025–1038. 10.1104/pp.17.01384 29138351 PMC5813575

[B82] Podolec R. Ulm R. (2018). Photoreceptor-mediated regulation of the COP1/SPA E3 ubiquitin ligase. *Curr. Opin. Plant Biol.* 45 18–25. 10.1016/j.pbi.2018.04.018 29775763

[B83] Pogson B. J. Ganguly D. Albrecht-Borth V. (2015). Insights into chloroplast biogenesis and development. *Biochim. Biophys. Acta Bioenerg.* 1847 1017–1024. 10.1016/j.bbabio.2015.02.003 25667967

[B84] Pokhilko A. Ramos J. A. Holtan H. Maszle D. R. Khanna R. Millar A. J. (2011). Ubiquitin ligase switch in plant photomorphogenesis: a hypothesis. *J. Theor. Biol.* 270 31–41. 10.1016/j.jtbi.2010.11.021 21093457 PMC3021735

[B85] Qi L. Liu S. Li C. Fu J. Jing Y. Cheng J. (2020). PHYTOCHROME-INTERACTING FACTORS Interact with the ABA Receptors PYL8 and PYL9 to Orchestrate ABA Signaling in Darkness. *Mol. Plant* 13 414–430. 10.1016/j.molp.2020.02.001 32059872

[B86] Qiu K. Li Z. Yang Z. Chen J. Wu S. Zhu X. (2015). EIN3 and ORE1 accelerate degreening during ethylene-mediated leaf senescence by directly activating chlorophyll catabolic genes in Arabidopsis. *PLoS Genet.* 11:e1005399. 10.1371/journal.pgen.1005399 26218222 PMC4517869

[B87] Reed J. W. Wu M. Reeves P. H. Hodgens C. Yadav V. Hayes S. (2018). Three auxin response factors promote hypocotyl elongation. *Plant Physiol.* 178 864–875. 10.1104/pp.18.00718 30139794 PMC6181040

[B88] Roig-Villanova I. Martínez-García J. F. (2016). Plant responses to vegetation proximity: a whole life avoiding shade. *Front. Plant Sci.* 7:236. 10.3389/fpls.2016.00236 26973679 PMC4770057

[B89] Rougemont J. Ljung K. Lo I. Solano R. Trevisan M. Pradervand S. (2012). Phytochrome interacting factors 4 and 5 control seedling growth in changing light conditions by directly controlling auxin signaling. *Plant J.* 1 699–711. 10.1111/j.1365-313X.2012.05033.x 22536829

[B90] Sakuraba Y. Jeong J. Kang M. Kim J. Paek N. Choi G. (2014). PIF4 and PIF5 induce leaf senescence in Arabidopsis. *Nat. Commun.* 5:4636. 10.1038/ncomms5636 25119965

[B91] Salisbury F. J. Hall A. Grierson C. S. Halliday K. J. Buildings K. Road M. (2007). Phytochrome coordinates Arabidopsis shoot and root development. *Plant J.* 50 429–438. 10.1111/j.1365-313X.2007.03059.x 17419844

[B92] Salome P. A. Xie Q. McClung C. R. (2008). Circadian timekeeping during early Arabidopsis development. *Plant Physiol.* 147 1110–1125. 10.1104/pp.108.117622 18480377 PMC2442538

[B93] Sampathkumar A. Eng R. C. (2020). Plant biology: bending of plant organs. *Curr. Biol.* 30 R402–R405. 10.1016/j.cub.2020.03.010 32369753

[B94] Sassi M. Lu Y. Zhang Y. Wang J. Dhonukshe P. Blilou I. (2012). COP1 mediates the coordination of root and shoot growth by light through modulation of PIN1- and PIN2-dependent auxin transport in Arabidopsis. *Development* 3412 3402–3412. 10.1242/dev.078212 22912415

[B95] Seluzicki A. Burko Y. Chory J. (2017). Dancing in the dark: darkness as a signal in plants. *Plant Cell Environ.* 40 2487–2501. 10.1111/pce.12900 28044340 PMC6110299

[B96] Sessa G. Carabelli M. Possenti M. Morelli G. Ruberti I. (2018). Multiple pathways in the control of the shade avoidance response. *Plants* 7:102. 10.3390/plants7040102 30453622 PMC6313891

[B97] Shin J. Kim K. Kang H. Zulfugarov I. S. Bae G. Lee C. H. (2009). Phytochromes promote seedling light responses by inhibiting four negatively-acting phytochrome-interacting factors. *Proc. Natl. Acad. Sci. U.S.A.* 106 7660–7665. 10.1073/pnas.0812219106 19380720 PMC2678665

[B98] Sibout R. Sukumar P. Hettiarachchi C. Holm M. Muday G. K. Hardtke C. S. (2006). Opposite root growth phenotypes of hy5 versus hy5 hyh mutants correlate with increased constitutive auxin signaling. *PLoS Genet.* 2:e202. 10.1371/journal.pgen.0020202 17121469 PMC1657057

[B99] Song Y. Yang C. Gao S. Zhang W. Li L. Kuai B. (2014). Age-triggered and dark-induced leaf senescence require the bHLH transcription factors PIF3, 4, and 5. *Mol. Plant* 7 1776–1787. 10.1093/mp/ssu109 25296857 PMC4261840

[B100] Soy J. Leivar P. González-schain N. Martín G. Diaz C. Sentandreu M. (2016). Molecular convergence of clock and photosensory pathways through PIF3 – TOC1 interaction and co-occupancy of target promoters. *Proc. Natl. Acad. Sci. U.S.A.* 113 4870–4875. 10.1073/pnas.1603745113 27071129 PMC4855547

[B101] Stepanova A. N. Robertson-Hoyt J. Yun J. Benavente L. M. Xie D. Y. Doležal K. (2008). TAA1-mediated auxin biosynthesis is essential for hormone crosstalk and plant development. *Cell* 133 177–191. 10.1016/j.cell.2008.01.047 18394997

[B102] Stepanova A. N. Yun J. Robles L. M. Novak O. He W. Guo H. (2011). The Arabidopsis YUCCA1 flavin monooxygenase functions in the indole-3-pyruvic acid branch of auxin biosynthesis. *Plant Cell* 23 3961–3973. 10.1105/tpc.111.088047 22108406 PMC3246335

[B103] Stephenson P. G. Fankhauser C. Terry M. J. (2009). PIF3 is a repressor of chloroplast development. *Proc. Natl. Acad. Sci. U.S.A.* 106 7654–7659. 10.1073/pnas.0811684106 19380736 PMC2678601

[B104] Toledo-Ortiz G. Johansson H. Lee K. P. Bou-Torrent J. Stewart K. Steel G. (2014). The HY5-PIF regulatory module coordinates light and temperature control of photosynthetic gene transcription. *PLoS Genet.* 10:e1004416. 10.1371/journal.pgen.1004416 24922306 PMC4055456

[B105] Vandenbussche F. Petrášek J. Žádníková P. Hoyerová K. Pešek B. Raz V. (2010). The auxin influx carriers AUX1 and LAX3 are involved in auxin-ethylene interactions during apical hook development in *Arabidopsis thaliana* seedlings. *Development* 137 597–606. 10.1242/dev.040790 20110325

[B106] Wabnik K. Abuzeineh A. Gallemi M. Van Der Straeten D. Zádníková P. Smith R. S. (2016). A model of differential growth-guided apical hook formation in plants. *Plant Cell* 28 2464–2477. 10.1105/tpc.15.00569 27754878 PMC5134968

[B107] Wang S. Zhang S. Sun C. Xu Y. Chen Y. Yu C. (2014). Auxin response factor (OsARF12), a novel regulator for phosphate homeostasis in rice (Oryza sativa). *New Phytol.* 201 91–103. 10.1111/nph.12499 24111723

[B108] Wang Y. Guo H. (2018). Tansley insight on hormonal regulation of the dynamic apical hook development. *New Phytol.* 222 1230–1234. 10.1111/nph.15626 30537131

[B109] Wang Z. Bai M. Oh E. (2012). Brassinosteroid signaling network and regulation of photomorphogenesis. *Annu. Rev. Genet.* 46 701–724. 10.1146/annurev-genet-102209-163450 23020777

[B110] Willige B. C. Ogiso-tanaka E. Zourelidou M. Schwechheimer C. (2012). WAG2 represses apical hook opening downstream from gibberellin and PHYTOCHROME INTERACTING FACTOR 5. *Development* 139 4020–4028. 10.1242/dev.081240 22992959

[B111] Wu G. Cameron J. N. Ljung K. Spalding E. P. (2010). A role for ABCB19-mediated polar auxin transport in seedling photomorphogenesis mediated by cryptochrome 1 and phytochrome B. *Plant J.* 62 179–191. 10.1111/j.1365-313X.2010.04137.x 20088903

[B112] Yang C. Li L. (2017). Hormonal regulation in shade avoidance. *Front. Plant Sci.* 8:1527. 10.3389/fpls.2017.01527 28928761 PMC5591575

[B113] Yu Y. Huang R. (2017). Integration of ethylene and light signaling affects hypocotyl growth in Arabidopsis. *Front. Plant Sci.* 8:57. 10.3389/fpls.2017.00057 28174592 PMC5258764

[B114] Žádníkova P. Petrášek J. Marhavý P. Raz V. Vandenbussche F. Ding Z. (2010). Role of PIN-mediated auxin efflux in apical hook development of *Arabidopsis thaliana*. *Development* 137 607–617. 10.1242/dev.041277 20110326

[B115] Žádníkova P. Wabnik K. Abuzeineh A. Gallemi M. Van Der Straeten D. Smith R. S. (2016). A model of differential growth-guided apical hook formation in plants. *Plant Cell* 28 2464–2477. 10.1105/tpc.15.00569 27754878 PMC5134968

[B116] Zhang X. Ji Y. Xue C. Ma H. Xi Y. Huang P. (2018). Integrated regulation of apical hook development by transcriptional coupling of EIN3/EIL1 and PIFs in Arabidopsis. *Plant Cell* 30 1971–1988. 10.1105/tpc.18.00018 30104405 PMC6181014

[B117] Zhang Y. Li C. Zhang J. Wang J. Yang J. Lv Y. (2017). Dissection of HY5/HYH expression in Arabidopsis reveals a root-autonomous HY5-mediated photomorphogenic pathway. *PLoS One* 12:e0180449. 10.1371/journal.pone.0180449 28683099 PMC5500333

[B118] Zhang Y. Liu Z. Chen Y. He J. Bi Y. (2015). Plant Science PHYTOCHROME-INTERACTING FACTOR 5 (PIF5) positively regulates dark-induced senescence and chlorophyll degradation in Arabidopsis. *Plant Sci.* 237 57–68. 10.1016/j.plantsci.2015.05.010 26089152

[B119] Zhong S. Shi H. Xue C. Wang L. Xi Y. Li J. (2012). A molecular framework of light-controlled phytohormone action in arabidopsis. *Curr. Biol.* 22 1530–1535. 10.1016/j.cub.2012.06.039 22818915 PMC4437768

[B120] Zhong S. Shi H. Xue C. Wei N. Guo H. Deng X. W. (2014). Ethylene-orchestrated circuitry coordinates a seedling’s response to soil cover and etiolated growth. *Proc. Natl. Acad. Sci. U.S.A.* 111 3913–3920. 10.1073/pnas.1402491111 24599595 PMC3964075

[B121] Zhu Q. Gallem M. (2019). Root gravity response module guides differential growth determining both root bending and apical hook formation in Arabidopsis. *Development* 146:dev175919. 10.1242/dev.175919 31391194

